# The omnipolar mapping technology—a new mapping tool to overcome “bipolar blindness” resulting in true high-density maps

**DOI:** 10.1007/s10840-023-01562-4

**Published:** 2023-05-25

**Authors:** Sebastian Dittrich, Cornelia Scheurlen, Jan-Hendrik van den Bruck, Karlo Filipovic, Jonas Wörmann, Susanne Erlhöfer, Jan-Hendrik Schipper, Jakob Lüker, Daniel Steven, Arian Sultan

**Affiliations:** Department of Electrophysiology, Heart Center, University of Cologne, Cologne, Germany

**Keywords:** High-density mapping, 3D mapping, Catheter ablation, Atrial fibrillation, Atrial tachycardia, Ventricular tachycardia

## Abstract

**Background:**

Omnipolar mapping (OT) is a novel tool to acquire omnipolar signals for electro-anatomical mapping, displaying true voltage and real-time wavefront direction and speed independent of catheter orientation. The aim was to analyze previously performed left atrial (LA) and left ventricular (LV) maps for differences using automated OT vs. standard bipolar settings (SD) and HD wave (HDW) algorithm.

**Methods:**

Previously obtained SD and HDW maps of the LA and LV using a 16-electrode, grid-shaped catheter were retrospectively analyzed by applying automated OT, comparing voltage, point density, pulmonary vein (PV) gaps, and LV scar area.

**Results:**

In this analysis, 135 maps of 45 consecutive patients (30 treated for LA, 15 for LV arrhythmia) were included. Atrial maps revealed significantly higher point densities using OT (21471) vs. SD (6682) or HDW (12189, *p* < 0.001). Mean voltage was significantly higher using OT (0.75 mV) vs. SD (0.61 mV) or HDW (0.64 mV, *p* < 0.001). OT maps detected significantly more PV gaps per patient vs. SD (4 vs. 2), *p* = 0.001.

In LV maps, OT revealed significantly higher point densities (25951) vs. SD (8582) and HDW (17071), *p* < 0.001. Mean voltage was significantly higher for OT (1.49 mV) vs. SD (1.19 mV) and HDW (1.2 mV), *p* < 0.001. Detected scar area was significantly smaller using OT (25.3%) vs. SD (33.9%, *p* < 0.001).

**Conclusion:**

OT mapping leads to significantly different substrate display, map density, voltage, detection of PV gaps, and scar size, compared to SD and HDW in LA and LV procedures. Successful CA might be facilitated due to true HD maps.

**Supplementary Information:**

The online version contains supplementary material available at 10.1007/s10840-023-01562-4.

## Introduction

Within the last decade, three-dimensional, electro-anatomical mapping systems have markedly improved, enabling a fast and thorough assessment of atrial and ventricular scars, tachycardia mechanisms, and gaps after previous catheter ablation (CA). This potentially facilitates consecutive CA of atrial and ventricular arrhythmia. Especially for ablation of scar-related or unstable ventricular tachycardia (VT), substrate-based ablation approaches have become the method of choice [1, 2].

**Fig. 1:**
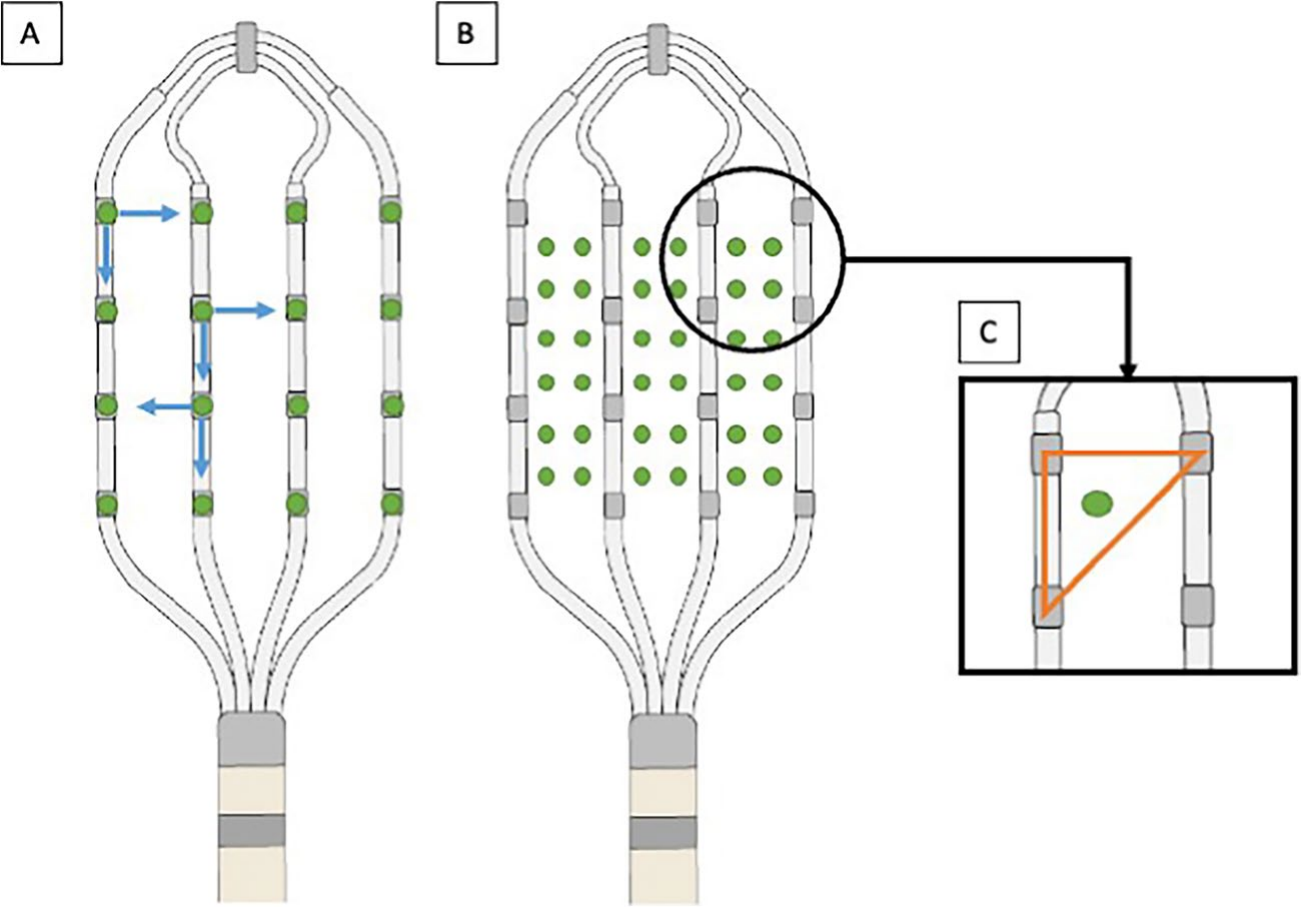
Electrode configuration and voltage points in HDW and OT configuration. Electrode configuration on the HD Grid catheter is displayed for HD wave Solution^TM^ (A) and Ensite^TM^ Omnipolar (**B**, **C**). **A** In HD wave configuration, the Best Duplicate algorithm choses the local bipole with the higher voltage. **B** In omnipolar configuration, local omnipoles are created that lead to a total of 36 mapping points. **C** Local omnipoles are created inside of a triangle formed by a clique of three adjacent electrodes

Standard bipolar mapping integrates only two electrodes at the same time, and the detection capability is limited to wavefronts along and across the electrodes. So, the standard bipolar mapping has its limitations in detecting wavefronts. Therefore, propagating wavefronts oblique to the catheter remain undetected (Fig. 1), a phenomenon also referred to as “bipolar blindness” [3, 4].

In contrast, the concept of a true high-density mapping is a simultaneous acquisition of several mapping points by using mapping catheters that feature more than two electrodes. Novel multipolar mapping catheters with particularly small interelectrode spacing allow fast and simultaneous acquisition of a much larger amount of mapping points.

One of these novel high-density mapping catheters is the *Advisor™ HD Grid Mapping Catheter* (*HD Grid*). It consists of 16 electrodes that are organized along 4 longitudinal splines, each spline featuring 4 electrodes. In a standard configuration, local bipolar signals are formed between any two electrodes in a longitudinal direction along the catheters’ splines (Fig. 1).

To overcome bipolar blindness, the *HD wave* (HDW) algorithm has been developed integrating an orthogonal bipole with the standard bipole [5, 6]. The *Best Duplicate* algorithm automatically selects the bipole with the higher voltage from these two adjacent pairs, lowering the effects of bipolar blindness (Fig. 1). Previous studies have shown that application of HDW with the *Best Duplicate* algorithm leads to higher voltages and better voltage delineation, especially in scar areas.

While adding an orthogonal bipole lowers the effect of bipolar blindness, true wavefront direction often follows a path in between the two available bipoles or changes rapidly and may therefore also remain undetected.

The *omnipolar technology* (OT) is a novel mapping tool used to acquire so-called omnipolar signals by integrating signals of three adjacent electrodes (a clique of three) that are organized in a triangular pattern [7]. In conjunction with the HD Grid catheter, OT mapping points are placed in the middle of these triangles, leading to 36 mapping points with a 2 mm spacing as compared to only 12 points with a 4 mm spacing using the HDW algorithm at one acquired mapping site [7] (Fig. 1). So therefore, using the same mapping catheter (HD Grid), OT maps display a higher resolution compared to standard bipole (SD) and HDW maps.

While traditionally, bipolar electrograms are local signals that do not contain information about directionality and speed, OT electrograms provide both. Thus, real-time data can be integrated into the Ensite^TM^ LiveView Dynamic Display module to visualize wavefront direction on a beat-to-beat basis in a real-time manner. Importantly, the triangle of electrodes acts as its own reference, thus omitting the need for an external reference.

In the present study, we retrospectively evaluate if the availability and usage of automated OT for electro-anatomical maps in the left atrium (LA) as well as the left ventricle (LV) would have led to different mapping outcomes in ablation procedures previously performed using HDW. We compare automated OT to SD and HDW settings, analyzing true voltage (*LA + LV*), point density (*LA + LV*), detection of pulmonary vein (PV) gaps per map (*LA*), and detected scar size (*LV*) for all three mapping modalities.

## Materials and methods

### Patient selection

This retrospective analysis consists of 135 maps of patients, who had been treated for CA of atrial or ventricular arrhythmia at the University Hospital Cologne, using the HDW mapping algorithm. In 30 consecutive patients, repeated CA for persistent atrial fibrillation (AF) as well as left atrial tachycardia (AT) had been performed. In fifteen consecutive patients, CA for VT or premature ventricular contractions (PVC) had been performed. Inclusion criteria were symptomatic persistent AF/AT/VT or PVC episodes, at least one prior ablation procedure (does not apply for VT/PVC group), and the usage of an HD Grid mapping catheter.

### Data analysis

In the course of the initial procedures, all maps were performed using HDW. These maps were converted into standard bipolar and OT maps afterwards and analyzed. So, for all patients, three maps (SD, HDW, and OT) were obtained from a single mapping procedure. Two independent analyses of baseline and procedural characteristics were performed for atrial and ventricular tachycardia, respectively. All obtained maps were analyzed and compared regarding voltage amplitudes and point density in OT vs. SD and HDW. Each patient acted as its own control in remapping the initial procedure with OT. Additionally, in all atrial procedures, each PV was analyzed for gaps (voltage and activation/propagation during SR), with a total of 120 pulmonary veins being mapped and evaluated for potential conduction gaps. In case of VT ablations, the scar area was assessed.

### Ablation procedure

All ablation procedures were performed under deep conscious sedation using propofol, midazolam, and fentanyl. A continuous oxygen insufflation and airway protection were established. At the end of the procedure and 2 h after, pericardial effusion was excluded using an echocardiogram.

All procedures were performed using the three-dimensional mapping system EnSite™ X, Abbott, Chicago, Illinois, USA. To obtain maps in LA or LV, an Advisor™ HD-Grid Sensor Enabled™ D- or F-curve, Abbott, USA, was used. In all procedures, the EGM acquisition was performed based on the following criteria: filtering criteria intracardiac ECG 30–300 Hz, surface ECG 0.5–50 Hz; geometrical interpolation 7; automatic acquisition criteria in line with Ensite AutoMap standard values.

#### Atrial fibrillation and atrial tachycardia

A preprocedural transesophageal echocardiography was performed to rule out left atrial thrombi. Oral anticoagulation was discontinued the day prior to the procedure. In patients on vitamin K antagonists, the procedure was performed within a therapeutic international normalized ratio range of 2.0–3.5.

Triple femoral venous access through the right femoral vein was performed, and a decapolar catheter was positioned in the coronary sinus. Afterwards, a fluoroscopy-guided single transseptal puncture was performed. Immediately after successful transseptal puncture, a weight-adjusted heparin bolus was administered, with repeat boluses every 30 min targeting an activated clotting time of > 300 s. For esophageal temperature monitoring, a temperature probe (*S-Cath, Esophageal Temperature Probe, Circa Scientific Inc., Englewood, Colorado, USA*) was orally placed in the esophagus.

Energy titration was guided by the lesion size index (LSI). For atrial ablation, the LSI target was 5.5 at the anterior and 4.5 at the posterior wall. The power setting for ablation was 40 watts.

#### Ventricular tachycardia

For VT ablation, at least one right femoral venous access was obtained for accessing the right ventricle, and at least one arterial access was established for invasive blood pressure management. Access to the left ventricle was obtained via transseptal access and/or retrograde aortic approach. In ventricular procedures, weight-adjusted heparin bolus was administered as well.

In most cases, a programmed ventricular stimulation was performed in advance to induce ventricular tachycardia. In some VT cases, pre-procedural cardiac CT or MRI images were integrated in the 3D mapping system to facilitate catheter ablation (Figure S1).

For ventricular ablation, the used power was between 30 and 60 watts depending on the localization and operator’s choice.

### Mapping protocol

**Fig. 2:**
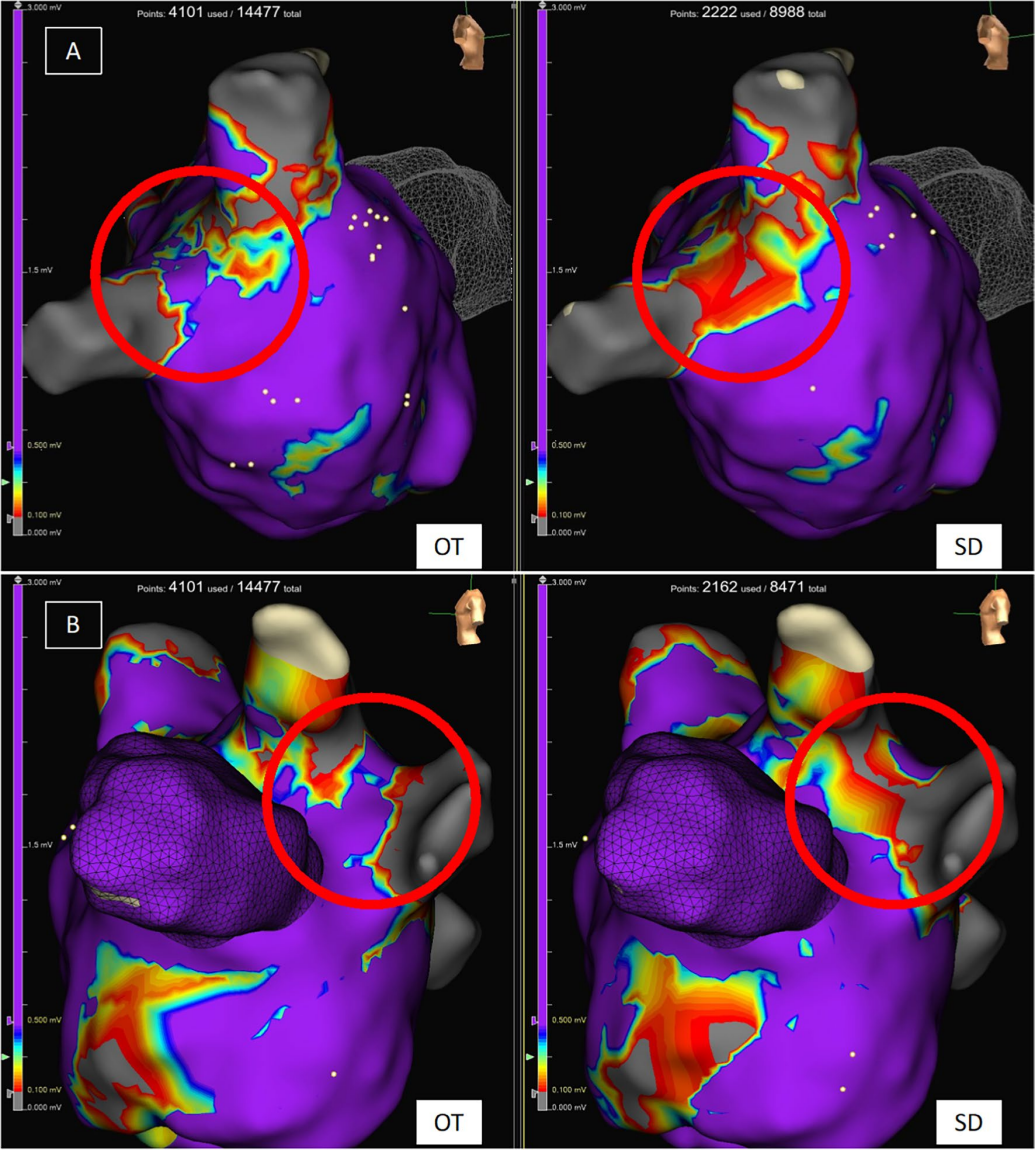
Substrate map of the LA in the area of PV gaps in OT and SD configuration. Atrial substrate maps of the LA displaying PV gaps around the right pulmonary veins (**A**) and left pulmonary veins (**B**). The displayed substrate maps have been performed using either OT settings (left) or SD settings (right). Scar threshold is set to 0.1 mV as used in many centers for demonstration purposes

All maps were performed in sinus rhythm. In the atrium, myocardial scar areas were defined by a voltage of < 0.2 mV as used in our center per standard protocol. For demonstration purposes and better comparability with other centers using lower scar threshold, we also provide Fig. 2 with exemplary substrate maps of PV gaps that have been mapped using a scar threshold of 0.1 mV. All PVs were analyzed in OT as well as in SD maps to detect possible gaps. A PV gap was defined by detectable voltage above mentioned thresholds and lack of PV entrance/exit block.

In ventricular maps, scar areas were defined by the number of points obtained with a voltage < 0.5 mV divided by the number of all collected points. Accordingly, the fraction of scar area was collected by dividing the number of mapping points with a voltage < 0.5 mV by the total number of voltage points in any of the maps. Potential VT isthmuses were confirmed by evaluation of stimulus to QRS onset, paced morphology, and presence of late abnormal ventricular activation (LAVA).

### Statistical analysis

Continuous data are displayed as mean and standard deviation and categorical variables as counts and percentages. For skewed data, median and interquartile range were used. Statistical significance was evaluated by students’ *t*-test and one-way repeated measures ANOVA. For comparison of two groups, student’s *t*-test was used. When three groups were compared, one-way repeated measures ANOVA was used. In these cases, for comparisons between any two of the included groups, Bonferroni-adjusted post-hoc analysis was performed. A *p*-value < 0.05 was considered statistically significant.

## Results

### Baseline characteristics

In total, 45 patients were included in this analysis, 30 with atrial and 15 with ventricular arrhythmia.

In atrial tachycardia, the mean age was 67.3 ± 9.0 years, and 13 (43%) were male. In 26 (87%) patients, CA was performed because of recurrent persistent AF and in 7 (23%) due to AT. Patients had undergone 1 [1-2] previous CA on average. The mean LA volume was 35.3 ± 10.2 ml, and the mean left ventricular ejection fraction was 55 ± 11%. The baseline characteristics of patients with atrial arrhythmia are shown in Table 1.

**Table 1:** Baseline characteristics in the atrial arrhythmia group

Baseline characteristics	Atrial arrhythmia
Patients, *n*	30
Age (years)	67.3 ± 9.0
Male gender, *n* (%)	13 (43%)
Previous ablation, *n* (%)	30 (100%)
Persistent AF, *n* (%)	26 (87%)
Atrial tachycardia, *n* (%)	7 (23%)
Comorbidities	
Arterial hypertension, *n* (%)	22 (73%)
Diabetes mellitus type II, *n* (%)	5 (17%)
Prior stroke, *n* (%)	7 (23%)
CAD, *n* (%)	2 (7%)
GFR (ml/min)	67.8 ± 20.6
BMI (kg/m^2^)	27.6 ± 6.1
OSA, *n* (%)	2 (7%)
CHA_2_DS_2_-VASc-score	4 (2–4)
LA volume (ml)	35.3 ± 10.2
LV-EF (%)	55 ± 11

Continuous data are summarized as means ± standard deviation or median (interquartile range), and categorial data are presented as number (percent)

*AF* atrial fibrillation, *BMI* body mass index, *CAD* coronary artery disease, *EF* ejection fraction, *LA* left atrium, *LV* left ventricle, *n* number, *OSA* obstructive sleep apnea

In ventricular tachycardia, the mean age was 61.6 ± 10.2 years, and 13 (87%) were male. Ablation procedures were performed for sustained ventricular tachycardia in 14 (93%) patients, while one (7%) patient received a PVC ablation. At least one prior ablation procedure has been performed in three (20%) patients. A history of cardiomyopathy was present in 15 (100%) patients, including an ischemic etiology (ICM) in 7 (47%) patients, dilated cardiomyopathy (DCM) in 7 (47%) patients, and arrhythmogenic right ventricular cardiomyopathy (ARVC) in 1 (7%) case. The mean left ventricular ejection fraction was 37 ± 12%. An implantable cardioverter defibrillator (ICD) was in place in 11 (73%) patients. One (7%) patient was carrying a left ventricular assist device (LVAD). Baseline characteristics of patients with VT are displayed in Table 2.

**Table 2:** Baseline characteristics in the ventricular arrhythmia group

Baseline characteristics	Ventricular arrhythmia
Patients, *n*	15
Age (years)	61.6 ± 10.2
Previous ablation, *n* (%)	3 (20%)
PVC ablation, *n* (%)	1 (7%)
VT ablation, *n* (%)	14 (93%)
Comorbidities	
Arterial hypertension, *n* (%)	9 (60%)
Diabetes mellitus type II, *n* (%)	3 (25%)
CAD, *n* (%)	9 (60%)
Cardiomyopathy, *n* (%)	15 (100%)
ICM, *n* (%)	7 (47%)
DCM, *n* (%)	7 (47%
ARVC, *n* (%)	1 (7%)
LVAD, *n* (%)	1 (7%)
ICD, *n* (%)	11 (73%)
GFR (ml/min)	75.8 ± 30.7
BMI (kg/m^2^)	29.6 ± 5.3
LV-EF (%)	37 ± 12

Continuous data are summarized as means ± standard deviation or median (interquartile range), and categorial data are presented as number (percent)

*AF* atrial fibrillation, *BMI* body mass index, *CAD* coronary artery disease, *EF* ejection fraction, *LA* left atrium, *LV* left ventricle, *n* number, *OSA* obstructive sleep apnea

### Procedural characteristics

#### Atrial fibrillation and atrial tachycardia

The overall procedure duration was 145 (120–168) min, fluoroscopy dose was 3253 (1654–5610) mGycm^2^, and fluoroscopy duration was 14.2 (12.3–20.2) min.

**Fig. 3:**
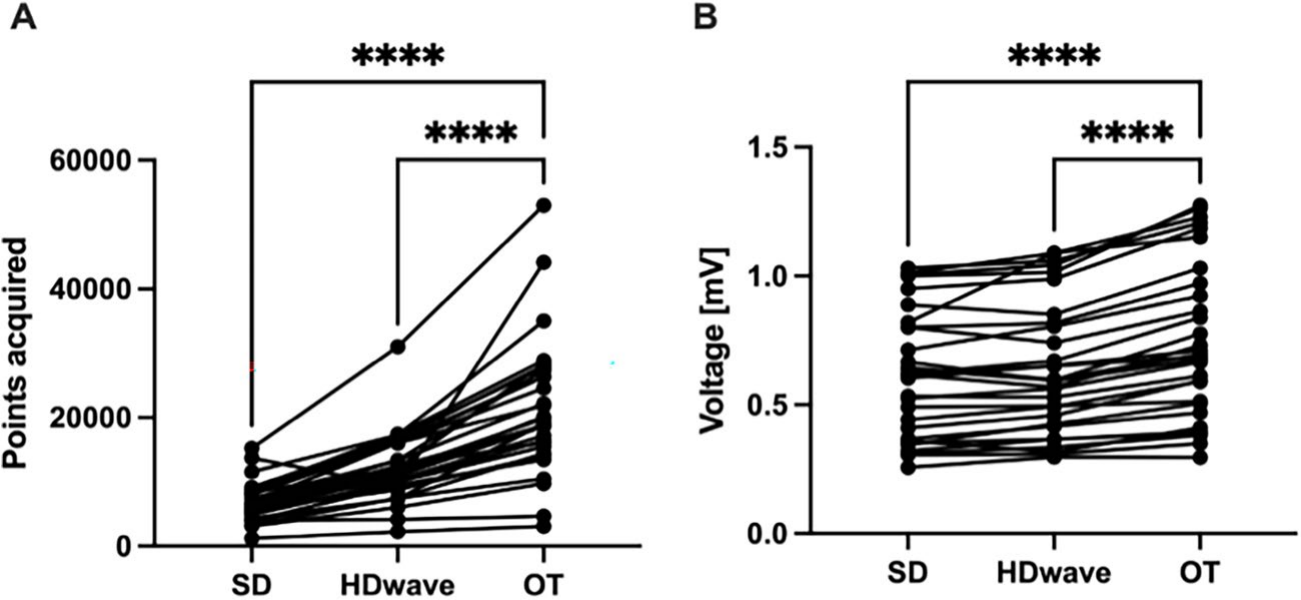
Mapping data of atrial maps. Line graphs displaying the amount of collected points (**A**) and voltage (**B**) of atrial maps for standard configuration (SD), HD wave, and omnipolar technology (OT). **** *p* < 0.0001

**Fig. 4:**
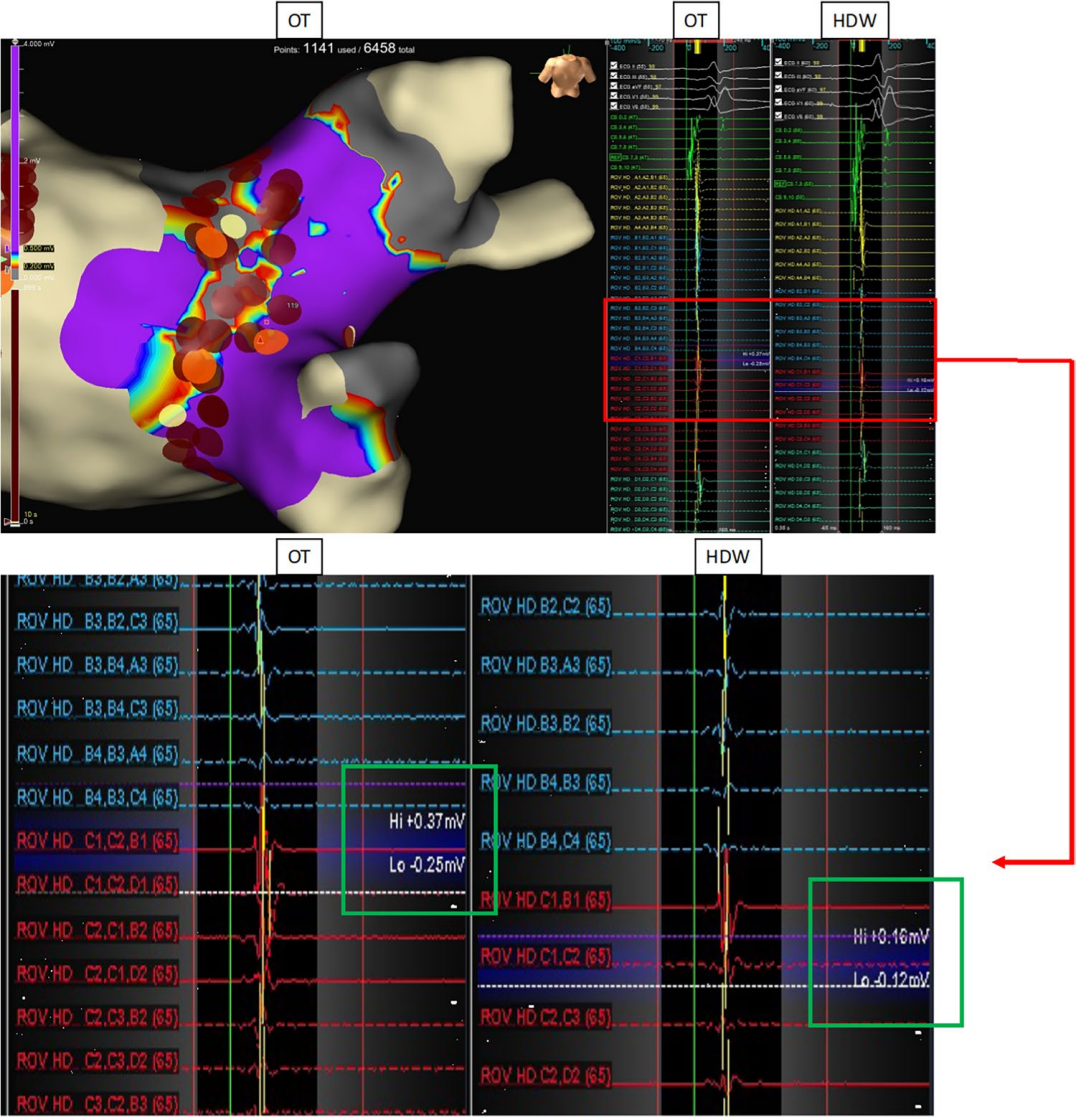
Substrate map of the LA in the area of a PV gap and associated EGMs. Substrate map of the LA at the site of a PV gap. The map has been performed using OT. The associated EGMs at the identified location of the PV gap are shown on the right (red box), calculated by OT and HDW. Amplitudes of the local EGMs around the gap site are higher in OT configuration vs. HDW configuration (0.62 mV vs. 0.28 mV), as visualized in the green boxes

Displayed LA mean voltage was significantly higher using OT as compared to SD as well as HDW, (OT: 0.75 ± 0.30, SD: 0.61 ± 0.24, HDW: 0.64 ± 0.26; *p*-value: SD vs. OT: < 0.001, HDW vs. OT: < 0.001, SD vs. HDW: 0.1). Also, obtained maps revealed a significantly higher point density using OT in comparison to the other mapping methods (OT: 21471 ± 10428, SD: 6682 ± 3009, HDW: 12189 ± 5346. *P*-values: SD vs. OT < 0.001, HDW vs. OT < 0.001, SD vs. HDW < 0.001) (Fig. 3). Furthermore, OT maps revealed significantly more PV gaps per map in comparison to standard maps (OT: 4 [3–5], SD: 2 [2-3], *p* = 0.001) (Table 2). Mapping data are shown in Table 3 and Fig. 3. Exemplary substrate maps of PV gaps using OT and SD configuration are displayed in Fig. 2, and exemplary EGMs inside the area of PV gaps are displayed in Fig. 4 for a more comprehensive visualization.

**Table 3:** Procedural characteristics in the atrial arrhythmia group

Procedural characteristics	SD	HD wave	Omnipolar	*p* value	
				SD vs. OT	HDW vs. OT
Voltage (mV)	0.61 ± 0.24	0.64 ± 0.26	0.75 ± 0.30	< 0.001*	< 0.001*
Points, *n*	6682 ± 3009	12189 ± 5346	21471 ± 10428	< 0.001*	< 0.001*
PV gaps (per map), *n*	2 (2–3)	-	4 (3–5)	< 0.001*	

*n* number, *PV* pulmonary vein, *SD* standard setting, *statistically significant

#### Ventricular tachycardia

The overall procedure duration was 210 (140–240) min, fluoroscopy dose was 7706 (3892–10867) mGycm^2^, and fluoroscopy duration was 22.1 (18.87–28.5) min.

**Fig. 5:**
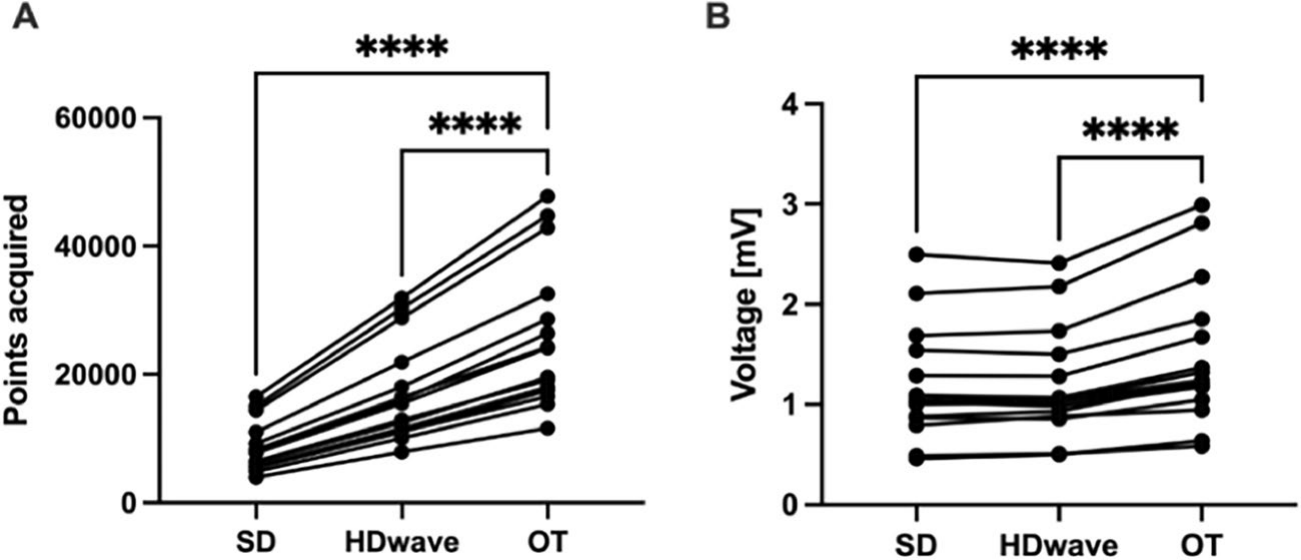
Mapping data of ventricular maps. Line graphs displaying the amount of collected points (**A**) and voltage (**B**) of ventricular maps for standard configuration (SD), HD wave, and omnipolar technology (OT). **** *p* < 0.0001

**Fig. 6:**
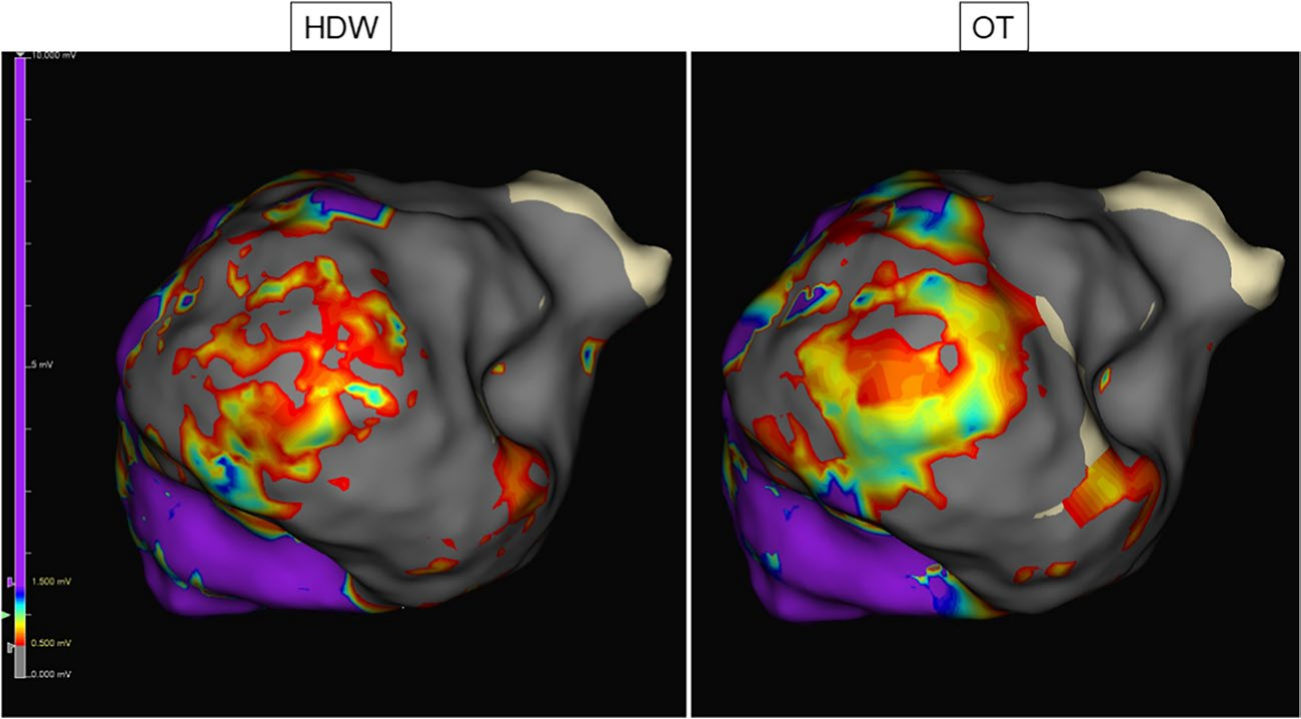
Substrate map of the posterior LV. Substrate mapping in the posterior LV using HDW and OT configuration. The OT map detects an area as viable myocardium that has been detected as scar in HDW configuration. The same voltage settings were used

Using OT, the displayed mean voltage was significantly higher as compared to SD and HDW (OT: 1.49 ± 0.72 mV, SD: 1.19 ± 0.56 mV, HDW: 1.2 ± 0.55 mV. *P*-values: SD vs. OT < 0.001, HDW vs. OT < 0.001, SD vs. HDW 1.0). Point density was also significantly higher in OT maps compared to SD and HDW (OT: 25951 ± 11340, SD: 8582 ± 3955, HDW: 17071 ± 7726. *P*-values: SD vs. OT < 0.001, HDW vs. OT < 0.001, SD vs. HDW < 0.001). The fraction of scar area was significantly smaller using OT compared to SD and smaller compared to HDW (OT: 25.3% ± 10.7%, SD: 33.9% ± 14.5%, HDW 29.8% ± 12.2%. *P*-values: SD vs. OT 0.007, HDW vs. OT 0.053, SD vs. HDW 0.002). Mapping data are displayed in Table 4 and Fig. 5. Exemplary substrate maps of the posterior LV scar area are displayed in Fig. 6.

**Table 4:** Procedural characteristics in the ventricular arrhythmia group

Procedural characteristics	SD	HD wave	Omnipolar	*p* value	
				SD vs. OT	HDW vs. OT
Voltage (mV)	1.19 ± 0.56	1.2 ± 0.55	1.49 ± 0.72	< 0.001*	< 0.001*
Points, *n*	8582 ± 3955	17071 ± 7726	25951 ± 11340	< 0.001*	< 0.001*
Scar area (%)	33.9 ± 14.5	29.8 ± 12.2	25.3 ± 10.7	0.007*	0.053

*n* number, *SD* standard setting, *statistically significant

## Discussion

### Main findings

The main findings in this study comparing LA and LV mapping using automated omnipolar technology vs. standard bipolar setting vs. HD wave are as follows:

1. Omnipolar technology achieved significantly higher voltage values in atrial as well as in ventricular arrhythmia.
2. Significantly higher point density was detected using omnipolar technology in atrial as well as in ventricular maps.
3. Significantly more PV gaps were detected using omnipolar technology.
4. Detected scar area was smaller using omnipolar technology in ventricular maps.
5. Therefore, all these findings enable a true high-density mapping potentially facilitating gap detection and CA for atrial and ventricular arrhythmia.

### Atrial fibrillation and atrial tachycardia

Arrhythmia recurrence after CA remains a challenge in modern electrophysiology. In case of pulmonary vein isolation (PVI) in the setting of AF, recurrence of AF is mostly attributed to the reconnection of PV gaps [8, 9]. Aside from improved CA techniques and approaches [9, 10], the limited sensitivity of current mapping tools to ensure PV isolation is considered a major reason for PV reconnection [8].

In our study, usage of OT led to a significantly higher mean voltage. As Masuda et al. [10] and Vlachos et al. [11] already showed, the presence and size of low-voltage areas predict recurrence of AF or AT after PVI. Since OT can measure the maximum voltage value independent of the catheter-wavefront orientation, it eliminates bipolar blindness [3] and potentially identifies real low-voltage areas more accurately than present mapping systems. The latter might be beneficial to improve the ablation approach by identifying real low areas in case of recurrence of AT after PVI.

Furthermore, using automated OT maps resulted not only in higher mean voltage but also in a significantly higher point density [6]. In line with our data, Papageorgiou et al. have already shown this finding using HDW compared to standard mapping [5].

Additionally, OT identifies statistically significant more PV gaps per map compared to the standard setting in our study. This is potentially crucial since, with standard mapping tools (circumferential mapping catheter), PV gaps might remain undetected, and therefore, the ablation strategy in these patients is debatable [12]. Papageorgiou et al. have previously evaluated the benefit of using the HDW algorithm to identify PV gaps as well. Also in this study, more PV reconnections could be identified using HDW [5]. As already mentioned, PV reconnection is the major reason for the recurrence of atrial fibrillation after PVI [8]. Hence, the higher mean voltage and higher point density could lead to a better identification of PV gaps, as demonstrated with local EGMs in Fig. 4. The use of HD mapping has led to varying scar thresholds in the LA and LV; additional exemplary images are demonstrated in Fig. 2, displaying advantages in the visualization of PV gaps also for a scar threshold of 0.1 mV. Identifying more PV gaps could significantly improve the freedom of atrial arrhythmia after ablation, especially in patients with recurrence of paroxysmal AF after previous PVI.

### Ventricular tachycardia

Ablation of scar-related VT relies on accurate substrate characterization. While cardiac MRI has become the gold standard for substrate visualization and characterization in the ventricles, clinical availability varies substantially between centers, and implementation into standard clinical workflow has proven challenging. Many VT patients are device carriers, further complicating usability in this patient cohort. Mapping algorithms like HDW and OT potentially close the gap between the accuracy of current mapping technologies and MRI (Figure S1). Previous studies have shown scar to correlate well with MR images using HD mapping techniques [13].

To our knowledge, we present the first OT evaluation in the context of VT ablation. In our study, the mean voltage was significantly higher in OT maps as compared to SD and HDW. This can be attributed to the effects of local omnipoles lowering the effect of bipolar blindness and assessing myocardial voltage independent of catheter orientation and wavefront direction. A true and high-density display of the actual scar is crucial in the context of scar-related VT ablation. As previous studies have shown, wavefronts inside scar areas tend to rapidly change directions of activation rather than following a linear course, aggravating the limitations that bipolar blindness imposes on conventional bipolar mapping techniques [4, 14, 15].

Application of OT led to maps of a significantly higher density compared to SD and HDW, measured by the number of points acquired. This is to be expected, with OT displaying 36 mapping points rather than 12 in the case of HDW, leading to a shorter spacing between mapping points (2 mm vs. 4 mm).

As previously described, benefits include the display of near-field potentials rather than far-field potentials, likely optimizing substrate visualization especially in low-voltage regions [5]. A shortening of the interelectrode spacing previously has been associated with better visualization of LAVAs, associated with arrhythmia occurrence and representing a frequent ablation target [16]. Studies performed with deceased human hearts have demonstrated that visualizations of wavefronts independent of catheter direction help in detecting LAVAs [17].

Importantly, the scar area was smaller in our study when using OT compared to SD or HDW, while not reaching statistical significance for OT vs. HDW. An exemplary substrate map of the posterior LV demonstrating better scar definition is displayed in Fig. 6. We hypothesize that this is a direct consequence of a more accurate substrate visualization provided by the local omnipoles. Additionally, by incorporating voltage, direction, and speed at the same time, OT might be more valuable. As substrate-based ablation approaches rely on substrate modification, visualization of remaining conduction channels in scar areas is crucial and a key to reducing arrhythmia recurrence [18, 19]. In practice, OT might thus help to guide the ablation procedure, render sites of interest unexcitable, possibly shorten procedure times, and consequently provide benefits in patient safety.

So far, OT has not been studied in the context of VT ablation, but Proietti et al. have evaluated HDW vs. SD in VT ablation procedures. Proietti reported obtaining maps with higher density and smaller scar sizes using HDW compared to SD, providing a better substrate definition especially at the scar border zone [5]. While in our case, scar size was smaller using OT vs. HDW, the results were not statistically significant. However, our study indicates that while the orthogonal vector in HDW helps mitigate the effects of catheter orientation on voltage, OT might provide additional, measurable benefits in substrate characterization and scar detection compared to HDW.

Furthermore, prospective studies are needed to evaluate if the application of OT has an impact on long-term ablation outcomes. MRI studies need to be performed to correlate substrate visualized by MRI and OT substrate mapping.

### Limitations

This study is a single-center retrospective analysis of previously obtained maps. OT was used in retrospect; prospective data are sparse. Additionally, there are no data regarding the outcome using OT in comparison to SD. Further studies need to be performed to assess a possible benefit on arrhythmia recurrence. Displayed data show that a potentially more precise scar definition using OT is likely. Furthermore, in our first cases, we observed a conclusive overlap with MRI and CT imaging. However, correlation of OT maps with MRI/CT and histological studies are to be performed in a larger scale in a prospective, randomized trial. However, the provided data underlines the significant difference using OT mapping. Therefore, it has been implemented in our clinical routine.

## Conclusion

In a subset of patients that have previously been treated by catheter ablation using substrate mapping by HDW, application of OT led to significant differences in substrate mapping results. Most importantly, OT provided a higher voltage and point density compared to SD and HDW resulting in more detected PV gaps. First data indicate a potential benefit for scar definition. Application of OT could provide a novel tool in improving substrate characterization for substrate-based ablation techniques especially in low-voltage areas and could ultimately lead to improvements in arrhythmia recurrence rates, procedure times, and procedural safety.

### Supplementary information

ESM 1 Figure S1: Ventricular substrate mapping using HDW (A) and pre-procedural cardiac MRI image (B), integrated in the 3D mapping system by ADAS software (Adas3D Medical S.L., Barcelona, Spain). Displayed scar area in substrate mapping correlate well with scar area in MRI image. (DOCX 1710 kb)

## Data Availability

All data and material are available upon contacting the corresponding author.
