# Supplementary material for: The omnipolar mapping technology—a new mapping tool to overcome “bipolar blindness” resulting in true high-density maps
Source: J Interv Card Electrophysiol. 2023 May 25;67(2):399–408. doi: 10.1007/s10840-023-01562-4 (PMC10901967; doi:10.1007/s10840-023-01562-4)

# Supplement

**Figure S1:**

Ventricular substrate mapping using HDW (A) and pre-procedural cardiac MRI image (B), integrated in the 3D mapping system by ADAS software (Adas3D Medical S.L., Barcelona, Spain). Displayed scar area in substrate mapping correlate well with scar area in MRI image.

**B**

**A**


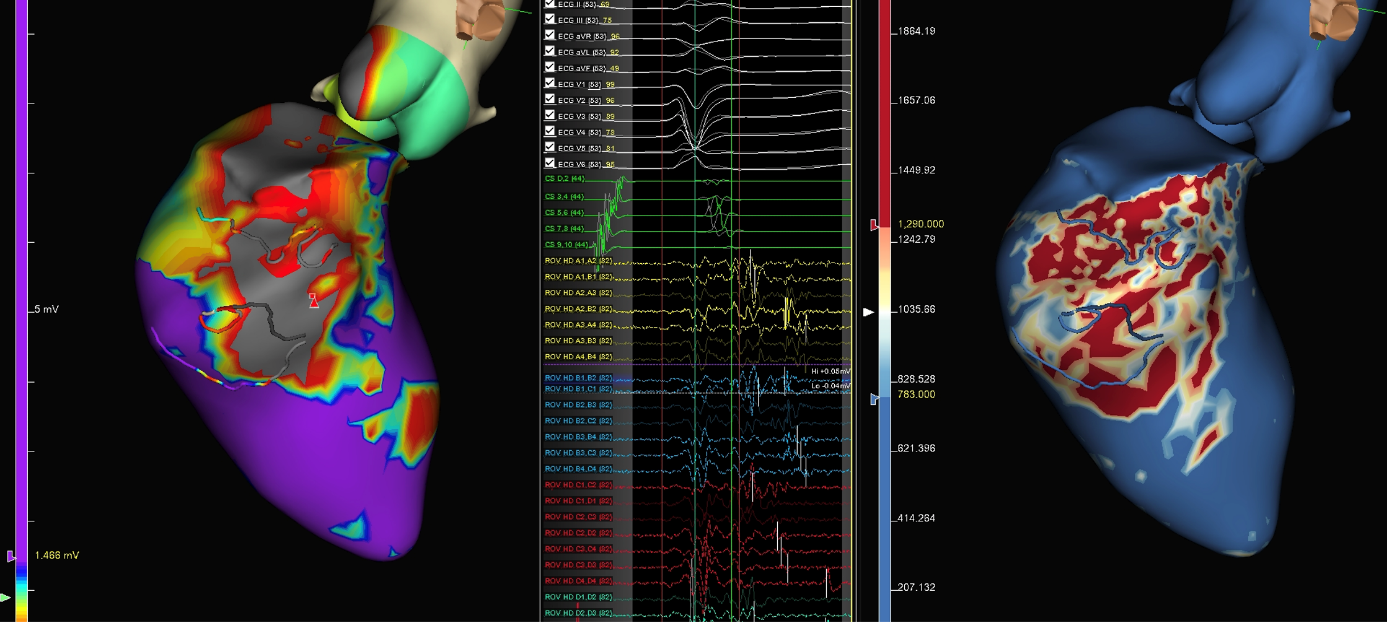

Supplement: Supplementary file 1 — Figure S1: Ventricular substrate mapping using HDW (A) and pre-procedural cardiac MRI image (B), integrated in the 3D mapping system by ADAS software (Adas3D Medical S.L., Barcelona, Spain). Displayed scar area in substrate mapping correlate well with scar area in MRI image. (DOCX 1710 kb) [file 10840_2023_1562_MOESM1_ESM.docx]
